# BMC medicine editorial board members on open access publishing

**DOI:** 10.1186/1741-7015-12-10

**Published:** 2014-01-21

**Authors:** Michael R Carmont, Stephen D Lawn, Babill Stray-Pedersen, Yehuda Shoenfeld, Pascal Meier

**Affiliations:** 1 Princess Royal Hospital, Shrewsbury and Telford NHS Trust and the Northern General Hospital, Sheffield Teaching Hospitals NHS Foundation Trust, Sheffield, UK; 2 Department of Clinical Research, Faculty of Infectious and Tropical Diseases, London School of Hygiene and Tropical Medicine, Keppel Street, London, UK; 3 Inst of Clinical Medicine, Oslo University and Div Women and children, Oslo University Hospital, Oslo, Norway; 4 Zabludowicz Center for Autoimmune Diseases, Sheba Medical Center (Affiliated to Tel-Aviv University), Tel-Hashomer 52621, Israel; 5 The Heart Hospital London, University College London Hospitals UCLH, London, UK

## Abstract

In recognition of Open Access week (21st-27th October 2013), we asked some *BMC Medicine* Editorial Board Members to share their views and experiences on open access publishing. In this short video, they highlight the benefits of visibility and dissemination of their research, and discuss the future directions for this model of publishing.

## 

## Supplementary Material

Additional file 1 BMC Medicine editorial board members talk on open access publishing. Click here for file

